# Extracting the Secrets of OpenSSL with RAMBleed

**DOI:** 10.3390/s22093586

**Published:** 2022-05-09

**Authors:** Chihiro Tomita, Makoto Takita, Kazuhide Fukushima, Yuto Nakano, Yoshiaki Shiraishi, Masakatu Morii

**Affiliations:** 1 Graduate School of Engineering, Kobe University, Kobe 657-8501, Japan; tomita.chihiro@gsuite.kobe-u.ac.jp (C.T.); zenmei@port.kobe-u.ac.jp (Y.S.); mmorii@kobe-u.ac.jp (M.M.); 2 Graduate School of Information Science, University of Hyogo, Kobe 651-2197, Japan; 3 Information Security Laboratory, KDDI Research, Inc., Saitama 356-8502, Japan; ka-fukushima@kddi-research.jp (K.F.); yuto@kddi-research.jp (Y.N.)

**Keywords:** rowhammer, RAMBleed, OpenSSL, side-channel attack, key recovery attack, RSA, Apache server

## Abstract

Concomitant with the increasing density of semiconductors, various attacks that threaten the integrity and security of dynamic random access memory (DRAM) have been devised. Among these, a side-channel attack called RAMBleed is a prolific one that utilizes a general user-level account without special rights to read secret information. Studies have reported that it can be used to obtain OpenSSH secret keys. However, a technique for deriving the Rivest–Shamir–Adleman (RSA) secret keys used in OpenSSL under realistic parameters and environments has not been reported. We propose a method that uses RAMBleed to obtain OpenSSL secret keys and demonstrate its efficacy using the example of an Apache server. The proposed method exploits the fact that, in the operation of an Apache server that uses OpenSSL, the RSA private keys are deployed on DRAM at a set time. Although the result of reading this secret information contains a few errors, error-free secret information is obtainable when it is used with RSA cryptanalysis techniques. We performed a series of attacks incorporating RAMBleed and eventually retrieved the OpenSSL RSA private key, indicating that secret information is obtainable with high probability. The proposed method can easily and externally be executed without administrator privileges on a server using DRAM that is vulnerable to RAMBleed, showing that RAMBleed is also a major threat to OpenSSL.

## 1. Introduction

With the rapid global rollout of 5G [1], various plans for leveraging the advantages offered by the technology are underway. This extends to the Internet of things (IoT), in which various types of data are acquired by sensors. As the data acquired by sensors include sensitive information such as data on human behavior and health conditions, it is necessary to use confidential communications for the exchange of such data [2,3]. In general, secret communication is achieved by encrypting data using a secret key. In this paper, we focus on the vulnerabilities of computing terminals and cryptographic implementations and demonstrate how secret keys can be stolen. An attacker who obtains a secret key can decrypt the associated encrypted data, making the communication insecure. Therefore, it is very important to analyze the security of communications in advance, considering a potential malicious attack.

To this end, we focus on a vulnerability in the dynamic random access memory (DRAM), which is invariably used in computing machines. Concomitant with the progressively increasing density of DRAM, the physical distance between the bits it contains is being reduced. This has spurred the occurrence of unintended bit flips, in which accessing one bit affects another bit—a behavior known as the rowhammer effect [4]. A rowhammer can be caused intentionally using software, and escalation [5], fault [6], and denial-of-service attacks [7] based on this phenomenon have been demonstrated. The target row refresh (TRR) function was developed as a countermeasure to provide resilience against rowhammer attacks; however, attacks such as SMASH [8], TRRespass [9], and Blacksmith [10] have since been devised to circumvent TRR and trigger the rowhammer effect. As rowhammer is a hardware-dependent vulnerability, it will persist until the vulnerable DRAM is replaced with a new DRAM featuring countermeasures, making it a significant problem that threatens computer-system security.

A rowhammer attack uses bit flipping to rewrite values stored in memory, thereby compromising the reliability of the data. These bit flips caused by rowhammer are affected by the value of the neighboring bits [4]. Considering this behavior, Kwong et al. developed the side-channel attack called RAMBleed to recover secret information without administrator access privileges by observing the bit flipping caused by an intentionally induced rowhammer [11]. Specifically, RAMBleed attempts to recover the secret information by sandwiching flippable bits in the secret information and exploiting the data dependence of the bit flips. For example, some flippable bits tend to flip from 1 to 0 when the bits above and below them are 0 but not when the bits above and below them are 1. In such cases, the secret value can be inferred to be 0 if the flippable bit is flipped and 1 if it is not flipped. Because the attacker does not have access to the secret, they must control the victim process to place the secret at the intended address to execute RAMBleed.

Kwong et al. showed that information on secret keys used by OpenSSH is obtainable using RAMBleed. Thus, as RAMBleed is a new attack that impairs the secrecy of data handled by computer systems, its impact must be fully analyzed.

It has been shown that the buddy allocator—a memory-allocation algorithm in Linux—can be used to place secret information [11]. However, to successfully place the secret information, it is necessary to analyze the order in which memory is allocated to the information in the victim process, and the attacker needs to predict, with high probability, when new physical memory will be allocated to the secret information. For OpenSSH, physical memory is newly allocated to the Rivest–Shamir–Adleman (RSA) private key when a child process created by the secure shell (SSH) daemon receives the transmission control protocol (TCP) connection and authenticates the client.

In this study, we investigated whether OpenSSL, which is used in numerous web servers, is secure against RAMBleed by devising a method for retrieving OpenSSL secret keys using RAMBleed. The method does not require administrator privileges. First, we investigated whether the data that comprises the RSA private key held on the server can be inducted into a specific memory location. To this end, we analyzed the behavior of a web server that employs the pre-fork model as a multiprocessing model. We discovered that when a newly created child process executes the transport layer security (TLS) handshake, physical memory pages are allocated to two prime numbers used to generate the RSA secret key. Moreover, we found that by leveraging the behavior of the pre-fork model to force the creation of a child process, there is a high probability that the TLS handshake can be performed with the new child process. Repeated observations of the timing of physical memory allocation to prime numbers revealed a bias in its timings. Subsequently, our experimental results showed that the two prime numbers used to generate the RSA secret key can be inducted with high probability into a specific address in the DRAM.

Based on the above results, we devised a series of attacks that involve reading a portion of an OpenSSL private key using RAMBleed and recovering the complete private key from the read information. Subsequently, we conducted experiments using the devised approach to recover two 1024-bit primes, *p* and *q*, constituting a 2048-bit RSA secret key. First, RAMBleed succeeded in reading each of the lower 570 bits with an accuracy of approximately 93%. Further, we succeeded in recovering all the bit values of the secret key from the lower 570 bits of each *p* and *q*, which were obtained using a combination of key recovery attacks against the RSA cipher, although the partial information contained a few percent errors [12,13]. Deriving RSA secret keys for OpenSSL under realistic parameters and environments has not been previously proposed. Our method is generally applicable to OpenSSL using RSA on servers fitted with DRAM that is vulnerable to RAMBleed and reveals that RAMBleed poses a significant threats to OpenSSL.

The remainder of this paper is organized as follows. Section 2 gives the details of the attack targets, OpenSSL, and Apache. Section 3 describes rowhammer and RAMBleed, which are the foundations of the proposed method. Section 4 describes the key-recovery attack against the RSA cipher used in the proposed method. In Section 5, we present our devised method for analyzing the behavior of Apache servers supporting secure sockets layer/transport layer security (SSL/TLS) communication using OpenSSL and demonstrate that OpenSSL secret keys are obtainable using RAMBleed. In Section 6, we present the proposed method for reading OpenSSL secret information. In Section 7, we demonstrate through reading experiments that the proposed method can recover RSA private keys in practice. Section 8 proposes countermeasures that may be useful against the proposed method and RAMBleed. Finally, Section 9 concludes this paper.

## 2. OpenSSL and Apache

This section describes the SSL/TLS communication that was the target of the attack in this study and used by the web server. This section also describes Apache, the open-source web-server software used to build the server.

### 2.1. SSL/TLS

SSL/TLS is a protocol for secure communication based on public key infrastructure (PKI). Encryption and authentication of communications using symmetric-key cryptography and public-key cryptography prevent eavesdropping, tampering, and spoofing. RSA is a public-key cryptosystem used for authentication. In authentication using RSA, a server certificate that links the public key to the server domain enables the client to verify if the party with whom they are communicating possesses the private key for the intended domain. A server certificate is issued by a certification authority based on a certificate signing request (CSR) created by the server administrator using a private key. Thus, if the private key is compromised, the certificate can be duplicated by a third party. RSA may be used to exchange the shared key for encrypted communications in TLS 1.2 and earlier. The information that forms the basis for the shared key is encrypted using the public key that corresponds to the private key held by the server and exchanged. Consequently, a third party possessing a compromised private key can intercept this exchange and duplicate the shared key. Thus, any leakage of private keys is crucial and compromises the security of the SSL/TLS protocol.

OpenSSL [14], developed as open-source software, is often used to build web servers that support SSL/TLS communication. OpenSSL implements the functionality of the latest version of the SSL/TLS protocol, TLS 1.3.

### 2.2. Apache

Apache [15] is an open-source web server software that is used globally. It can be used with OpenSSL to construct a web server that supports hypertext transfer protocol secure (HTTPS) communications. Three different multiprocessing models are implemented in Apache to handle simultaneous requests from multiple clients. One of these is the pre-fork model, which dynamically adjusts the number of processes based on the number of requests. In the pre-fork model, the server-parent process spawns several child processes in advance and assigns requests to idle child processes. If the parent process receives more requests than the number of idle processes, it responds by creating new child processes. Additionally, it is necessary to limit the number of idle processes to control memory consumption because memory is allocated to each child process in the pre-fork model.

In Apache, the minimum and maximum number of idle processes are defined by MinSpareServers and MaxSpareServers, respectively. If the number of idle processes exceeds the MaxSpareServers value, the parent process terminates the extra idle child processes; if it falls below the MinSpareServers value, a new child process is created.

## 3. RAMBleed

This section explains the structure of DRAM and Linux memory management and describes rowhammer, on which RAMBleed is based. Subsequently, we explain the use of RAMBleed in this study.

### 3.1. DRAM

**Figure 1:**
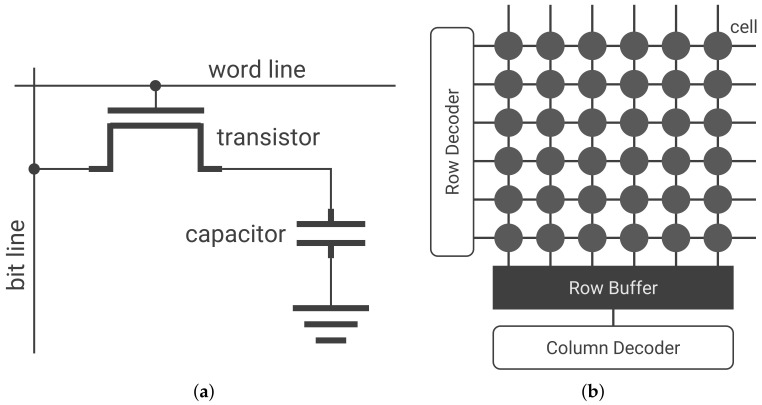
Structure of dynamic random access memory (DRAM): DRAM consists of cells that hold one bit of information. By increasing the word line voltage, one line of information is loaded into the row buffer; the CPU accesses the row buffer to read and write data. (**a**) Structure of a cell: each cell is comprised of a capacitor and a transistor. (**b**) Structure of a bank: cells are arranged in matrix form.

DRAM holds one bit of information in a cell, composed of a capacitor and a transistor, as shown in Figure 1a. The value of the bit is determined by whether the capacitor is charged, and access to the cell is controlled by the transistor. A cell that represents one and zero in the charged and uncharged states, respectively, is called a true cell. Conversely, a cell that represents zero and one when charged and uncharged is called an anti-cell. For simplicity, this study only considers true cells. Cells are arranged in the form of a matrix, with each row connected by word lines and each column by bit lines as shown in Figure 1b. A fixed number of rows are collectively called a bank, and a DRAM chip is composed of multiple banks. Multiple DRAM chips are collectively referred to as a rank and are mounted on one side of a dual inline memory module (DIMM).

The data in DRAM are handled in rows of 8 KiB. These data are accessed by increasing the voltage of the word line of the row in which they are located; a capacitor is connected to the bit line. This process, known as activating a row, transfers one line of data to the row buffer, as shown in Figure 1b. Subsequently, the CPU accesses the target column from the data in the row buffer and reads/writes data. The charge stored in the DRAM cell capacitor is gradually lost over time. Thus, it is crucial to periodically recharge the capacitor to prevent data loss: this operation is termed a refresh. A refresh is performed by periodically activating each row.

### 3.2. Linux Buddy Allocator

**Figure 2:**
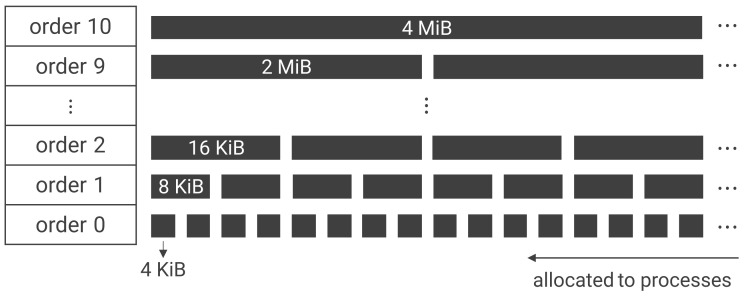
Memory management in Linux via the buddy-allocator algorithm. Memory spaces are divided into orders numbered 0 to 10. Each order is further subdivided into blocks, with the blocks in different orders having different sizes. When a process requests memory allocation, the buddy allocator allocates the smallest block for the required area. Only order 0 requests are allowed from user space.

Linux manages the system memory in units called pages, which have a minimum size of 4 KiB each. The buddy-allocator algorithm is used to allocate and deallocate these pages. The kernel classifies all free memory space into order 0–10 as a single block for each physically contiguous page. Figure 2 shows that, order *n* consists of blocks of $2^{n}$ pages, and the memory blocks in each order have a stack-like first-in-last-out (FILO) data structure. When a process requests memory allocation, the buddy allocator allocates the smallest block for the required area. However, only order 0 requests are allowed from user space. For example, if a user process requests a 12 KiB memory boundary, this is processed as three 4 KiB requests. Thus, when a memory allocation is requested from user space, the blocks in order 0 are allocated first, regardless of the size of the request.

### 3.3. Rowhammer

Rowhammer is a phenomenon in which repeated access to multiple rows of DRAM induces bit flips in neighboring rows [4]. This phenomenon can be exploited to rewrite data without direct access to the memory region we desire to invert, which is a major problem that threatens the safety of computer systems. More specifically, the explanation for this process is as follows. As the cell density increases, the word lines become more closely packed. Consequently, when the voltage of one word line is increased, it causes the voltage of neighboring word lines to slightly increase, resulting in the loss of charge from the cell at that location. This effect can be magnified by activating the same row multiple times within a short period; if sufficient charge is lost to exceed the noise margin before a refresh takes place, a bit flip occurs.

The ease of inducing a rowhammer bit flip depends on the values of the bits in the rows above and below the bit. Specifically, bit flipping is most likely to occur when there are uncharged cells above and below a charged cell [4]. For example, suppose that the values of three adjacent bits in the same column are denoted from top to bottom as x−y−z. In true cells, cells with a bit value of one are charged. Therefore, the 0−1−0 state is the most prone to bit flipping. In this state, repeated access to the rows containing the upper and lower bits may cause a reversal of the middle bit, changing it to 0−0−0. However, the 1−1−1 state is less likely to experience bit flipping, and is more likely to remain 1−1−1 despite repeated access.

Several DDR3 DRAM modules experience rowhammer-induced bit flipping. There are various tools to verify if installed DRAM is susceptible to rowhammer [4,5].

### 3.4. RAMBleed

Kwong et al. proposed RAMBleed, which leverages the rowhammer effect to breach the secrecy of data [11]. In the RAMBleed attack, the attacker exploits the dependency of bit flipping by rowhammer to predict the value of the bits immediately above and below it with high probability. This enables the attacker to read information such as secret keys stored in memory areas that are not directly accessible. The attacker does not require privileges because it is sufficient for the attack to allocate free memory and to access memory areas owned by the attacking process.

**Figure 3:**
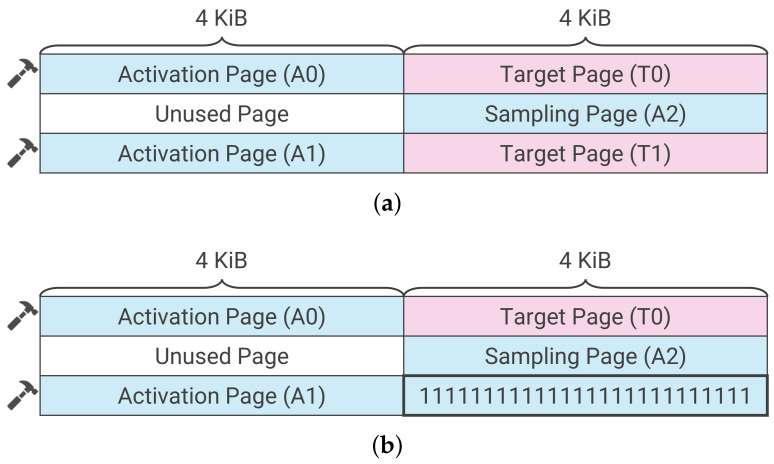
Memory layouts used for RAMBleed. The layouts consist of activation, target, and sampling pages. The attacker inducts secrets into target pages and repeatedly accesses pages A0 and A1 to activate the pages. The rowhammer effect causes the bits in the sampling page to invert, and the attacker can infer the secret by observing it. (**a**) Memory layout used by Kowng et al. for RAMBleed. (**b**) Simplified memory layout used in this study for RAMBleed.

RAMBleed is comprised of two phases: a preparation phase, in which a memory layout is created and bit flipping is observed, and an attack phase that positions the secret information and reads it. Kwong et al. considered the memory layout shown in Figure 3a, inducted secret information at T0 and T1 in the target page, and proposed a method for inferring secret information using the dependency of bit flipping. The induction of secret information is not always successful in environments where various processes run together on a server because secret information may only be placed on either T0 or T1. In this case, the read accuracy is lower than that when the secret information is placed at both T0 and T1. Additionally, the positioning of the secret information within the page must be perfectly aligned. Therefore, it is difficult to use the layout that requires the secret information to be placed in exactly two locations, as shown in Figure 3a.

Consequently, RAMBleed was performed using the memory layout shown in Figure 3b in this study. The target page is set to one location on T0, and secret information is directed to one location. Although the number of bit positions to be bit-flipped is reduced compared to that when the secret is placed in two locations, the accuracy of the read remains almost the same. Additionally, because RAMBleed does not allow the user to specify the bit positions to be restored, repeated attacks may read the same bit position multiple times. In this case, if the bit value is determined by majority vote, the read accuracy can be improved. Consequently, we describe below the RAMBleed procedure using the simplified layout.

#### 3.4.1. Creating a Memory Layout and Searching for Bit Inversion

The attacker first allocates three adjacent rows of physical memory, as in the conventional RAMBleed approach [11]. Subsequently, all bit values in the bottom right page are set to one, as shown in Figure 3b. Each page is 4 KiB in size, and each row contains two pages. Here, the areas indicated by A0, A1, and A2 in the figure are the pages allocated to the process of the attacker, and the area indicated by T0 is the page allocated to the target process.

All bit values in T0 are set to zero and all bit values in A2 are set to one when searching for bit positions to use in the attack stage. Subsequently, the A0 and A1 pages are repeatedly accessed and the rowhammer attack is executed. Transfers to the row buffer in DRAM are performed in rows, such that if an attacker accesses page A0, two pages of data (A0 and T) are transferred to the row buffer. Therefore, bit flipping can be induced within A2. The attacker is able to read and write data and verify whether a bit flip has occurred because A2 is a page allocated to the process of the attacker. The attacker records the bit positions at which the bit flips occurred and uses them to recover the secret information in the attack phase.

#### 3.4.2. Inducting Secret Information

The attacker launches the process to be attacked and directs it to the target page (T0), which contains the secret information they desire to read. A method called Frame Feng Shui (FFS), which uses the buddy allocator, is used for induction [11]. This method assumes that the attacker knows the number of physical memory pages (dummy pages) to be allocated before the target process accesses the secret information.

**Figure 4:**
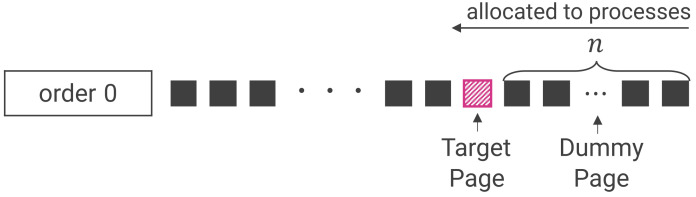
Allocation of secret information. The target page is the page where the secret to be recovered by RAMBleed is to be stored, and the dummy pages are pages reserved for data to be accessed by the attack target before the secret. When the attack begins, the buddy allocator assigns the dummy pages to the attack target first. The secret is then assigned to the target page.

**step 1 (Reserve memory pages)**.  Assume that, after it is launched, the process to be attacked stores the desired secret information on page n+1. At this time, the attacker reserves an area for *n* pages;
**step 2 (Release memory pages)**.  The attacker releases the page (target page) on which they want the secret information to be placed. Subsequently, all *n* pages allocated in step 1 are released. Consequently, there is an area for *n* pages at the top of order 0 of the buddy allocator, with the target page immediately after it, as shown in Figure 4;
**step 3 (Launch the process to be attacked)**.  Immediately after step 2, the attacker launches the target process, which allocates the first *n* pages of order 0 and stores the secret information in the target page.

#### 3.4.3. Reading Secret Information

Here, we explain a method for reading secret information using the data dependency of bit inversion. We denote the *i*th bit (i∈{0,1,...,32767}) from the beginning of page P as P[i]. The attacker identifies the position where the bit flip occurs in A2 in the preparation phase, and we assume that the bit flip occurs at A2[i]. We assume that the secret information is successfully inducted and is contained in T0.

First, all the bit values in A2 are set to one. Next, the attacker repeatedly accesses A0 and A1 and triggers a rowhammer attack on A2. Finally, the attacker checks the value of A2[i]. If T0[i] is one, this single-column is 1−1−1; therefore, bit flipping is unlikely to occur in A2[i]. Conversely, if T0[i] is zero, this column is 0−1−1; therefore a bit flip is likely to occur at A2[i], resulting in 0−0−1. Thus, there is a high probability that T0[i] and A2[i] match following the rowhammer attack. Consequently, an attacker can read some of the information by confirming the value of A2[i] with high probability.

## 4. Algorithm for Recovering RSA Private Keys

If the secret information is successfully inducted, it can be read with high accuracy using RAMBleed, albeit with several percentage-point errors. This section describes two existing attacks against the RSA cryptosystem, that can be used to correct the reading data.

### 4.1. Paterson et al.’s Algorithm

The algorithms presented by Heninger et al. [16], Henecka et al. [17], and Paterson et al. [12] can be utilized to recover the correct private key from a corrupted secret key. Prime numbers *p* and *q*, which constitute the RSA private key under attack are read using RAMBleed, contain different proportions of bits that should be zeros but are ones, and vice versa. The manner in which this error occurs is similar to the error model assumed in the algorithm proposed by Paterson et al. This algorithm takes the erroneous *p* and *q* as input and reconstructs the most likely $\hat{p}$ and $\hat{q}$ in groups of *t* bits beginning from the least significant, using the following process. The likelihood measure is defined as the probability that an error occurs at a given rate of bit reversal in a candidate series that matches the input series. Paterson et al. calculated this likelihood by determining the bit-flip error rates from 0 to 1 and from 1 to 0 using α and β, respectively.

**step 1**. Make empty sequences $\tilde{p}$ and $\tilde{q}$;
**step 2**. Extend $\tilde{p}$ by *t* bits and determine $\tilde{q}$ using a relational expression that must be satisfied by the public key, *p* and *q*. Thus, $2^{t}$ pairs of $\left(\tilde{p},\tilde{q}\right)$ are candidates;
**step 3**. Calculate the likelihood of each candidate;
**step 4**. Retain *L* candidates in descending order of likelihood. Execute step 2 for the remaining *L* candidates.

This operation is repeated until the required number of bits is obtained, and *L* candidate secret keys ($\hat{p}$, $\hat{q}$) with the highest likelihood are selected. The larger the values of α and β, the larger the values of *t* and *L* are required, which increases the time required for restoration, and decreases the accuracy of restoration.

### 4.2. Coppersmith’s Methodology

Coppersmith showed that when $(\log_{2}N)/4$ bits are obtained from the least significant bit of *p* or *q*, all bits of each prime can be recovered [13]. Let $p_{0}$ be the known lower $(\log_{2}N)/4$-bit value of prime *p* and *k* be the number of unknown bits. We can find the value of f(x) that satisfies the value of *p* by finding the solution of $f\left(x\right)=x^{k}+p_{0}=0\;\left(\mathrm{mod}\;p\right)$. Coppersmith’s theorem states that for any integer *n* and monic polynomial (a polynomial such that the highest order coefficient is one) f(x), any $|x|<n^{1/d}$ satisfying f(x)=0(modN) can be found in feasible time, as can *p* [13].

Coppersmith’s method is implemented using SageMath [18], a Python-based system for processing mathematical equations. The small_root function in SageMath can find the solution of an equation for divisor *b* of *N* such that $b≧N^{\beta }$; therefore, it can easily implement Coppersmith’s method. Since *N* is the product of *p* and *q*, which have the same number of bits, we can make b=p or *q* a law by setting β=0.5.

## 5. Analysis of OpenSSL and Apache

In this study, we targeted an Apache server that uses OpenSSL. Specifically, we used RAMBleed to read the information making up the RSA private key stored on the server. Secret information must be inducted in the manner described in Section 3.4.2 for RAMBleed to succeed. This method assumes that the attacker knows the number of dummy pages. This section analyzes the behavior of the Apache server, which supports SSL/TLS communication via OpenSSL, and provides a method for deriving the number of dummy pages in advance. The results of the experiment show that the number of dummy pages can actually be predicted with high probability.

### 5.1. Experimental Environment

We used a server running Ubuntu 18.04 on a core i5-4590 CPU with two DDR3 4 GiB 1333 MHz non-ECC DIMMs. An Apache2.4.46 server was configured to support TLS communication using OpenSSL1.1.1. We issued a self-certificate using the private key created by the user and registered it in the configuration file of the server. The pre-fork model was assumed as the multiprocessing model of the server. All other settings were left at the default values.

### 5.2. Investigating Whether Physical Memory Is Allocated for Secret Information

This section describes our method for investigating whether physical memory pages are allocated to secret information following the start of the web-server process, i.e., when a TLS request is received from a client, and presents the results of this investigation. The secret information considered are two 1024-bit prime numbers, *p* and *q*, used to generate the RSA private key.

#### 5.2.1. Identifying the Virtual Addresses of Secret Information

In the pre-fork model, child processes are created by the fork-system call. Immediately after a fork is executed, the parent and child processes share the same virtual memory using copy on write (COW) to suppress unnecessary memory consumption. However, when a child process executes a write operation on a virtual memory page, the corresponding page in the shared virtual memory is duplicated, and only the child process can access that page. This is because a page fault occurs, and the kernel allocates a new physical memory page as no physical memory page is allocated for the duplicated virtual memory page.

We focused on this behavior and analyzed it, theorizing that secret information is written to virtual memory when the child process performs the TLS handshake and that a physical memory page allocation occurs. To simplify the analysis, MaxSpareServers was set to one; therefore, there was always one idle child process, which limits the number of child processes communicating. When the TLS handshake was executed, the SSL/TLS version was specified by the client as TLS1.3 and the cipher suite as AES256-GCM-SHA384. The investigation was conducted using the following procedure.

**step 1**. Obtain a virtual memory dump, A, of the idle child process using the gcore command;
**step 2**. Send a TLS request and conduct a TLS handshake with only one child process. After the handshake, a virtual memory dump, B, of the child process conducting the TLS communication is obtained;
**step 3**. Search virtual memory dumps A and B, and identify the virtual address of the secret information. If there is a virtual address for secret information that exists only in B, we know that the secret information with that address was written to the virtual memory of the child process when the TLS handshake was executed.

The above analysis was conducted both on a child process that had performed the TLS handshake after its creation and a child process that had not. The virtual addresses of *p* and *q* did not change before and after the TLS handshake, and no new secret information was written for the child process that had already executed the TLS handshake. Conversely, we discovered that new prime numbers *p* and *q* were written to virtual memory after the TLS handshake was executed for the child process that had not executed the TLS handshake; we could identify their virtual addresses.

#### 5.2.2. Physical Memory Allocation to Prime Numbers

We also examined whether the physical addresses of the pages containing *p* and *q*, confirmed to be written to virtual memory, change before and after the TLS handshake. A change in the physical address indicates that physical memory has been newly allocated to *p* and *q*. The physical address of a virtual memory page to which no physical memory is allocated is zero.

It is crucial to refer to the page table that stores the virtual address of each virtual memory page of the server process and its corresponding physical address to verify the physical address allocated to given information. In a Linux kernel built with CONFIG_PROC_MONITOR=y, the page table of a process can be accessed from /proc/(process ID of the target process)/pagemap with administrator privileges. The investigation revealed that physical memory was not allocated to *p* and *q* before the TLS handshake was executed and that it was newly allocated after executing the handshake. Considering the results in Section 5.2.1as well, it is confirmed that physical memory is allocated to *p* and *q* when the child process performs the TLS handshake for the first time.

#### 5.2.3. Identifying the Writing of Prime Numbers in Memory

In this section, we present the method used to identify the process that allocates physical memory to *p* and *q* and the results of the analysis of the server process that performs the TLS handshake with the client. The value of Apache’s MinSpareServers was set to one to simplify the analysis. The GNU project debugger (GDB) was used for the analysis. An overview of the the analysis method is provided below.

First, we started the Apache server and performed a TLS handshake once with the server-child process. Next, we identified the virtual addresses of *p* and *q* from the virtual memory dump of that process. A new child process was created because the number of idle child processes was less than the MinSpareServers value. The new child process has not yet performed the TLS handshake. We attached to this process with GBD and set watchpoints at the the virtual addresses of *p* and *q*. In this state, the process that was stopped for the GDB to be attached was restarted, multiple TLS requests were sent externally, and the attached process received one of the requests. The process that received the TLS request stops when it accesses the watchpoint during execution of the TLS handshake. Following that, we verified the data indicated by the watchpoint. When that data are neither *p* nor *q*, we restart the process. This operation is repeated until *p* and *q* appear in the virtual memory at the point indicated by the watchpoint. Finally, we identified the process that accessed *p* and *q* in OpenSSL by observing the stack trace at the time *p* and *q* appeared in memory.

The analysis revealed that in the TLS1.3 handshake, *p* and *q* are written to the physical memory page in the process of composing the CertificateVerify message sent from the server to the client. The CertificateVerify message is used to prove that the server possesses the private key corresponding to the public key and includes a digital signature created by the private key of the server. We discovered that access and physical memory allocation to *p* and *q* occurs in the OpenSSL function that is responsible for the digital signature process. Moreover, rather than allocating physical memory to *p* and *q* simultaneously for access, they are written to pre-allocated physical memory pages during the TLS handshake. Furthermore, after the server-child process executes the TLS handshake, *p* and *q* remain written in the physical memory allocated to the child process for as long as that child process exists. However, when the number of child processes on the server exceeds the MaxSpareServers value, the server-child processes are destroyed by the parent process, and *p* and *q* may be purged from the memory simultaneously. Therefore, it is necessary to maintain communication with the child process to keep *p* and *q* in the physical memory page.

Considering the above analysis, an attacker can cause *p* and *q* to be written to the target page by releasing the dummy pages and the target page before the TLS handshake is executed. Additionally, these can be read using RAMBleed by maintaining communication with the server-child process because *p* and *q* remain in the target page after the TLS handshake is executed.

#### 5.2.4. How to Perform a TLS Handshake with a New Child Process

For the physical memory page allocation to *p* and *q* to occur in the actual process of communication, the attacker must cause the server to create child processes and perform a TLS handshake with one of the newly created child processes. In this section, we describe a method for achieving this by utilizing the behavior of the pre-fork model. The procedure is as follows.

**step 1 (Send MaxSpareServers TCP requests)**.  The server has a maximum of MaxSpareServers idle processes. Therefore, when a MaxSpareServers TCP request is sent, the server allocates the request to existing idle child processes, and if there are insufficient child processes, it spawns child processes until MinSpareServers processes are idle. This enables at least MinSpareServers new child processes to be created. The client that causes the creation of these new child processes is called a dummy client;
**step 2 (Send a TLS request)**.  In Apache, a parameter called timeout defines the amount of time a child process waits between receiving a TCP packet from a client and then receiving the next TCP packet, during which time the child process is in a non-idle state. Unless a dummy client sends a FIN packet, the existing child process will not become idle; therefore, the only idle process on the server for the time set by Timeout will be the new child process. If an attacker sends a TLS request within the duration set by Timeout, there is a high probability that a TLS handshake can be executed with the new child process. When a parent process creates and terminates multiple child processes, it must wait at least 1s after sending a request from a dummy client before sending a TLS request because only one process is handled per second.

**Figure 5:**
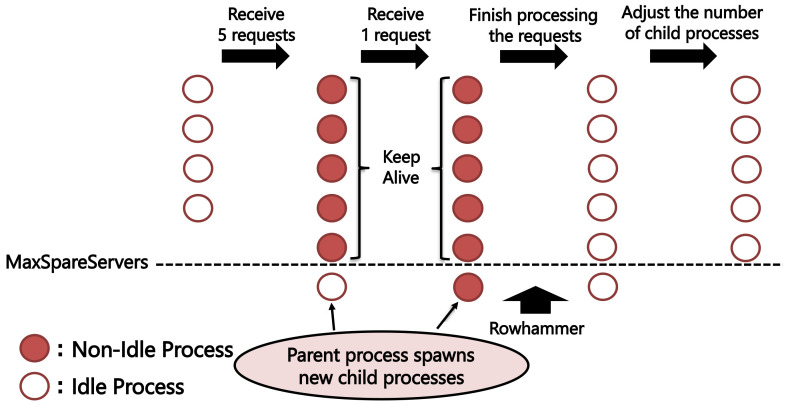
Process by which the server-parent process creates new child processes. When the number of non-idle processes running is equal to the MaxSpareServers value, the server-parent process creates a new child process for an additional request.

Using this method, the attacking process can perform a TLS handshake with the newly created child process and generate physical memory allocations for *p* and *q* with high probability. Figure 5 illustrates how the server creates a new child process when MaxSpareServers=5 and MinSpareServers=1.

### 5.3. Determination of Dummy Page Count

Physical memory can be newly allocated to *p* and *q* in the manner described in Section 5.2. We determined the number of dummy pages required to induct *p* and *q* empirically. In the following experiment, MaxSpareServers was set to the default value of ten.

#### 5.3.1. Determination Method

The number of dummy pages, where the ID of the process that owns the target secret information and the virtual address of that secret information within the virtual address space of that process are known, can be determined using the following five steps.

**step 1**. Obtain 100 physical memory pages and record the physical address of each page;
**step 2**. Release each of the pages obtained in step 1 in turn and record them in order at order 0 in the buddy allocator for each page. Record the order of the released pages with the last released page first because the released pages are managed in a stack data structure on order 0;
**step 3**. Send a MaxSpareServers request to the server, and then a TLS request one second later;
**step 4**. Determine the physical address of the page containing the secret information from the process ID of the server process with which the measurement process is communicating and the virtual address of the secret information;
**step 5**. The number of dummy pages is equal to one less than the number of pages released in step 2 whose physical address matches the physical address of the secret information at order 0.

Repeat the five-step process above to ascertain the number of dummy pages and observe the bias in the number of dummy pages. Notably, the virtual addresses of *p* and *q* are consistent between child processes owing to COW. The virtual address of the virtual memory page replicated by writing *p* and *q* is the address in the virtual memory shared with the parent process. Therefore, if each child process writes *p* and *q* to the same virtual memory page during the TLS handshake, their virtual addresses do not change between the child processes.

#### 5.3.2. Experiment to Determine the Dummy Page Count

Table 1 shows the results of 100 runs of a measurement program that performs the five steps above for prime number p. The results of measurement showed a large bias in the number of dummy pages, with 35 pages being the most common, being measured 84% of the time. Table 2 shows the results of the measurements for prime number *q*. For *q*, there was a similar bias in the number of pages measured, with 43 pages being measured 81% of the time. Because there is a large bias in the number of dummy pages for both primes *p* and *q*, if the number of dummy pages is set based on these values, *p* or *q* can be inducted using the method described in Section 3.4.2, and their information can be partially read using RAMBleed. A possible cause for the two measured values is that one of the 100 pages released from the measurement process was allocated to a process other than the process performing the TLS handshake.

## 6. Reading OpenSSL Secret Information

In this section, we present our proposed method that uses RAMBleed to retrieve the OpenSSL secret information used on Apache servers. The secret information to be attacked includes the two *n*-bit prime numbers *p* and *q* that compose the RSA secret key; the attack procedure is presented below.

**step 1**. Repeat RAMBleed until the required number of bits is obtained, and read the secret information in memory;
**step 1-1**. In the sampling page, create a layout where bit flipping occurs at the position where the prime numbers are placed.;
**step 1-2**. Induct secret information into the target page;
**step 1-3**. Execute rowhammer on the layout and read the bit value at the position where the bit flip occurred in the sampling page in step 1;
**step 2**. Input the bit string into the algorithm presented by Patterson et al. with the value of the bit position that could not be read in step 1 set to one. This returns the partial secret information;
**step 3**. Recover all bit values from the partially recovered secret information using Coppersmith’s algorithm.

In the following section, we describe the preparations required to execute the proposed method and how each step is performed in detail.

### 6.1. Preparing the Attack

For RAMBleed to succeed, secret information must be inducted in the manner described in Section 3.4.2. This method assumes that the attacker knows the number of dummy pages. The number of dummy pages is obtained using the method presented in Section 5.2. The information to be read by RAMBleed is determined by back-calculating the partial information required to recover the secret using Coppersmith’s method.

### 6.2. Partially Reading Secret Information Using RAMBleed

In Step 1, RAMBleed reads part of the secret information. The attack procedure is similar to that described in Section 3.4; however, when targeting OpenSSL, the attacker must pay attention to the following.

It is sufficient to search for bit inversions in the range where *p* and *q* are written in the sampling page because prime numbers *p* and *q* are written to specific locations in the virtual address space of all server-child processes. When inducing secret information, it is equally necessary to perform a TLS handshake with the newly created server-child process in the manner described in Section 5.2.4. However, when the server processes a request received before the TLS request, the dummy page or target page released by the attacker may be purged. Therefore, it is necessary to release the dummy and target pages immediately before sending a TLS request.

The number of bit inversions depends on the DRAM. Additionally, there are some areas where the server process cannot search for inversions. If an attacker cannot detect enough bit flips to recover secret information, the following contrivances can be used. The physical memory area to be searched can be changed by restarting the server and changing the memory allocated to each process. In the course of the experiment, we confirmed that the bias in the number of dummy pages is likely to remain unchanged owing to server restarts. The attacker can prompt a server to restart through a denial of service (DoS) attack or a distributed denial of service (DDoS) attack. Moreover, both true cells and anti cells exist in the DRAM. Therefore, if the search for inverted bits in true cells does not yield a sufficient number of inverted bits, more inverted bits can be found by switching the search target to anti cells.

### 6.3. Recovering Secret Information

Since the partial secret information read by RAMBleed contains errors, it is corrected in step 2 using the algorithm presented by Patterson et al.. Finally, in step 3, we use Coppersmith’s method to recover all bit values of the secret information from the uncorrupted partial secret information. If the public key can be recovered from the recovered private key, the attack has succeeded. Depending on the error conditions, error correction using the algorithm proposed by Patterson et al. may not always be successful. If it fails, we use RAMBleed to take readings and then repeat the operation to correct errors again by increasing the number of bits read or by decreasing the number of error bits included in the information that is read.

## 7. Experiment Using RAMBleed to Read an RSA Private Key

In this section, we describe an experiment conducted that supports SSL/TLS communication via OpenSSL to read secret information on an Apache server, following the attack procedure set out in the previous section. In the experiment, the secret information of the attack target included the 1024-bit prime numbers, *p* and *q*, that compose the 2048-bit RSA secret key held by the server. The experimental environment was the same as in Section 5.1.

### 7.1. Preparing the Attack

Using the method presented in Section 5.3, we determined the number of dummy pages required to induct *p* and *q* to the layout. Since the experimental environment was the same, the measured results were the same as those in Table 1 and Table 2. A large bias was observed in the number of dummy pages, with 35 pages for *p* and 43 pages for *q* being the most frequently measured. Considering that there is a large bias in the number of dummy pages for both primes *p* and *q*, if the number of dummy pages is set based on these values, *p* or *q* can be inducted using the method described in Section 3.4.2, and their information can be partially read using RAMBleed.

A preliminary experiment was conducted using Coppersmith’s method to determine the amount of information that must be read using RAMBleed. The results show that if the lower 570 bits of either of the two 1024-bit primes can be obtained, all the bit values of the two primes can be recovered. Therefore, in step 1, the lower 570 bits of prime numbers *p* and *q* should be read using RAMBleed. Errors resulting from this can be corrected using the algorithm presented in Paterson et al.

### 7.2. Reading an RSA Private Key Using RAMBleed

The lower 570 bits of *p* and *q* were targeted, and step 1 was repeated over six weeks for each *p* and *q*, enabling overlap in the bit positions to be read. During the read period, the server was restarted multiple times and the read target switched from true cells to anti cells to find the inverted bit in more bit positions. In the experiment, the server was restarted by the administrator without using a DoS attack.

Table 3 shows the results of *p* and *q* readings. The number of bits read in the experiment is the number of bits read (with duplicates). RAMBleed may read the same position, and if a position is read more than once, RAMBleed adopts the result of the majority of readings. The number of bit positions with duplicates removed in this manner is called the number of read bits (no duplicates). Accuracy is the percentage of bits with correct values among the bit values (without duplicates) read using RAMBleed. The false positive rate is the percentage of bits that are 1, but incorrectly read as 0, in *p* and *q*. The false negative rate is the percentage of bits that are 0,but incorrectly read as 1, in *p* and *q*. The probability that rowhammer can correctly reflect the value of any one bit of the target page on the sampling page was approximately 95%, and the accuracy of induction to the target page was approximately 83%. These results indicate that the accuracy with which one bit of inducted information is read once is approximately 79%. However, both *p* and *q* were ultimately read with high accuracy because the proposed method reads the same bit position multiple times. Server restart indicates the number of times the server was restarted during readings of the true and anti cells.

It was impossible to read all lower bits, even after restarting the server and switching between true and anti-cells. This may be due to the presence of cells that do not physically invert in the DRAM. As the number of read bits increased, the probability of reading a value at a new nonoverlapping bit position decreased; therefore, reading was terminated when acceptable reading accuracy was achieved using the algorithm proposed by Patterson et al.

### 7.3. Recovering Secret Information

All values of the lower bits of prime numbers *p* and *q* were determined by setting all bit values that could not be read using RAMBleed to one. The bit-flip error rates from 0 to 1 and from 1 to 0 were α=0.0974 and β=0.0403, respectively. Using the noisy *p* and *q* as inputs and setting the parameters to t=20 and L=10, the algorithm proposed by Paterson et al. yielded the error-free lower 570 bits of *p* and *q*. Moreover, Coppersmith’s algorithm was successfully applied to recover the secret key by using the lower bits of *p* or *q* as input and recovering all the bit values of either *p* or *q*. These experimental results showed that the RSA private key used in OpenSSL, which does not require administrator privileges, can be recovered from information read by RAMBleed.

We further investigated the extent to which the algorithm succeeds when the error rate is the same as that in the present experiment, but with different locations of errors. This is because the success of the algorithm proposed by Paterson et al. depends on the occurrence of errors. The results of the experiment to recover the secret key from a bit string with errors at random bit positions for *p* and *q* in the proportions α=0.0974 and β=0.0403 showed that recovery was successful ten times out of 100. If the recovery failed, we continued reading the secrets using RAMBleed. Since the false positive rate and false negative rate are small, the same bit can be read multiple times to correct the false bits by majority voting. Reducing errors in the read result in this way will make it easier to succeed when attempting to restore again.

## 8. Discussion

In this study, we demonstrated that RAMBleed can be used to obtain RSA secret information from an Apache server that supports SSL/TLS communication using OpenSSL. The two prime numbers that constitute the RSA private key held by the server were read and recovered without administrative privileges. Based on the process and results of the reading experiment, we provide the following guidelines for devising measures against the proposed method.

If the OS assigns the pages released from the attacking process during the induction of secret information to a server-child process other than the one communicating with the attacking process, induction will fail. Therefore, running processes that frequently consume physical memory in addition to the server process in the server can reduce the accuracy of induction.

The number of dummy pages may differ from the values shown in this study based on the configuration and version of Apache or OpenSSL. Where the number of dummy pages is unknown, the attacker must repeat the attack while adjusting the number of dummy pages specified when RAMBleed is executed. In the experiments, it took a long time to read *p* and *q* to obtain sufficient accuracy for recovery. Therefore, the risk of recovery can be reduced by frequently updating the server’s secret key.

The proposed method is effective because prime numbers *p* and *q* remain in physical memory changebyowing to the server process during the TLS handshake. RAMBleed reads can be avoided by removing *p* and *q* from memory immediately after the process that uses them in the TLS handshake is completed.

## 9. Conclusions

The increasing density of DRAM has increased the feasibility of rowhammer attacks, witch cause bit flipping in memory. RAMBleed uses rowhammer to indirectly read data in memory and is a major threat because it does not require administrator privileges and can read secret information held on a computer with high accuracy. In this study, we executed RAMBleed on an Apache server that supports SSL/TLS communication using OpenSSL and demonstrated that RAMBleed can read the secret information held by the server. Using these results, we implemented and tested a series of attacks involving RAMBleed and succeeded in recovering prime numbers.

In this study, we demonstrated that it is possible to retrieve secret information used in OpenSSL, for which no method has hitherto been proposed, revealing a major threat to OpenSSL. Although the DRAM used in this study does not feature any TRR countermeasure, a new rowhammer attack that overcomes TRR has been devised for DRAM. Attacks against this kind of DRAM can be made using a new rowhammer attack in combination with the proposed attack method. Therefore, it is necessary to consider countermeasures not only from a hardware perspective, but also from an OpenSSL implementation perspective.

## Figures and Tables

**Table 1:** Dummy page-count measurements for *p*.

Number of Dummy Pages	Number of Observations
35	84
36	16

**Table 2:** Dummy page-count measurements for *q*.

Number of Dummy Pages	Number of Observations
43	81
44	19

**Table 3:** Reading prime numbers *p* and *q* using RAMBleed.

	*p*	*q*
Accuracy	96.25%	95.32%
False Positive	2.20%	6.03%
False Negative	5.36%	3.17%
Number of bits read (with duplicates)	6689	6831
Number of bits read (without duplicates)	534	534
Server restarts (true cells)	4 times	7 times
Server restarts (anti cells)	0 times	4 times

## Data Availability

Not applicable.
